# Supplementary material for: Molecular Subtypes as a Basis for Stratified Use of Neoadjuvant Chemotherapy for Muscle-Invasive Bladder Cancer—A Narrative Review
Source: Cancers (Basel). 2022 Mar 26;14(7):1692. doi: 10.3390/cancers14071692 (PMC8996989; doi:10.3390/cancers14071692)
Supplement: Supplementary file 1 [file cancers-14-01692-s001.zip › cancers-1632458 supplementary_proofs_220330.pdf]

cancers

MDPI

Supplementary Material

# Molecular Subtypes as a Basis for Stratified Use of Neoadjuvant Chemotherapy for Muscle-Invasive Bladder Cancer—A Narrative Review

**Gottfrid Sjödahl, Johan Abrahamsson, Carina Bernardo, Pontus Eriksson, Mattias Höglund and Fredrik Liedberg**

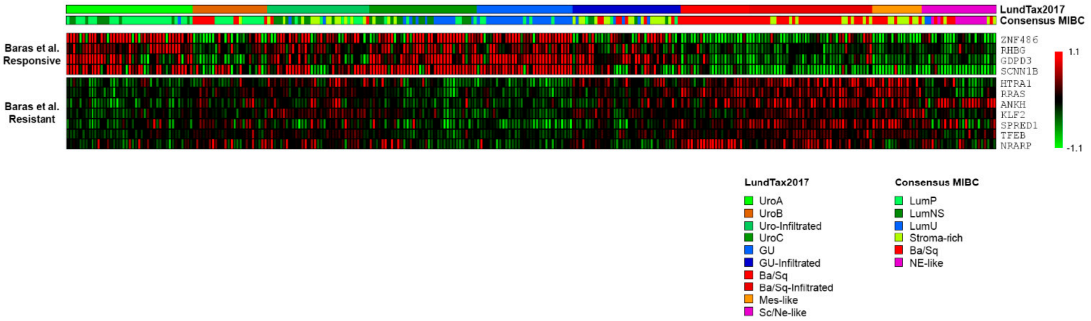


**Figure S1.** Genes associated with NAC response/resistance in Baras et al. [48] are expressed in luminal and Basal/squamous MIBC subtypes, respectively. The 21 genes shown in Figure1 of Baras et al. [38] identified as associated with NAC response were investigated in the Lund2017 consecutive RC cohort gene expression data set ($n$ = 307). All mapped genes in the responsive cluster were highly expressed in luminal-like subtypes, whereas all mapped genes in the Baras et al. Resistant cluster were highly expressed in the Ba/Sq subtype.

**Table S1.** Fraction of chemotherapy response in Taber et al. [20] ($n$ = 121), stratified by consensus subtypes, treatment setting, and DDR-mutations. Number of cases with a potentially predictive mutation (ERCC2, BRCA2, or any DDR-mutation) or wild-type for all three mutation types, is shown separately for responders treated with NAC (left) or first-line only (right). Based on manual review of source data from Taber et al [20]. The last columns show the fraction of responders with presence of at least one of the three potentially predictive mutation types.

**Chemotherapy response by consensus subtype, treatment setting, and DDR-mutation status in Taber et al. 2020**

| Consensus Subtype | NAC-Response | ERCC2 | BRCA2 | DDR | 3xWT | 1L only-Response | ERCC2 | BRCA2 | DDR | 3xWT | % of responses explained by DDR-status |
|---|---|---|---|---|---|---|---|---|---|---|---|
| LumP | 9/15 (60%) | 1 | 2 | 1 | 7 | 21/29 (72%) | 3 | 3 | 8 | 12 | 37% (NAC: 22%, 1L only: 43%) |
| LumU | 8/10 (80%) | 2 | 2 | 2 | 5 | 10/15 (66%) | 1 | 2 | 5 | 4 | 50% (NAC: 37%, 1L only: 60%) |
| Stroma-rich | 6/7 (86%) | 2 | 2 | 5 | 1 | 7/11 (64%) | 0 | 1 | 0 | 6 | 46% (NAC: 83%, 1L only: 14%) |
| BASQ | 6/12 (50%) | 3 | 2 | 3 | 2 | 9/20 (45%) | 1 | 1 | 3 | 5 | 53% (NAC: 67%, 1L only: 44%) |
| NE-like | - | - | - | - | - | 1/1 (100%) | 0 | 0 | 0 | 1 | 0% (1L only: 0%) |
